# WGDdetector: a pipeline for detecting whole genome duplication events using the genome or transcriptome annotations

**DOI:** 10.1186/s12859-019-2670-3

**Published:** 2019-02-13

**Authors:** Yongzhi Yang, Ying Li, Qiao Chen, Yongshuai Sun, Zhiqiang Lu

**Affiliations:** 1 CAS Key Laboratory of Tropical Forest Ecology, Xishuangbanna Tropical Botanical Garden, Chinese Academy of Sciences, Mengla, 666303 Yunnan China; 2 State Key Laboratory of Grassland Agro-Ecosystem, College of Life Sciences, Lanzhou University, Lanzhou, China

**Keywords:** Whole genome duplication, dS, Genome, Transcriptome

## Abstract

**Background:**

With the availability of well-assembled genomes of a growing number of organisms, identifying the bioinformatic basis of whole genome duplication (WGD) is a growing field of genomics. The most extant software for detecting footprints of WGDs has been restricted to a well-assembled genome. However, the massive poor quality genomes and the more accessible transcriptomes have been largely ignored, and in theoretically they are also likely to contribute to detect WGD using dS based method. Here, to resolve these problems, we have designed a universal and simple technical tool WGDdetector for detecting WGDs using either genome or transcriptome annotations in different organisms based on the widely used dS based method.

**Results:**

We have constructed WGDdetector pipeline that integrates all analyses including gene family constructing, dS estimating and phasing, and outputting the dS values of each paralogs pairs processed with only one command. We further chose four species (*Arabidopsis thaliana*, *Juglans regia*, *Populus trichocarpa* and *Xenopus laevis*) representing herb, wood and animal, to test its practicability. Our final results showed a high degree of accuracy with the previous studies using both genome and transcriptome data.

**Conclusion:**

WGDdetector is not only reliable and stable for genome data, but also a new way to using the transcriptome data to obtain the correct dS distribution for detecting WGD. The source code is freely available, and is implemented in Windows and Linux operation system.

**Electronic supplementary material:**

The online version of this article (10.1186/s12859-019-2670-3) contains supplementary material, which is available to authorized users.

## Background

Polyploidy or whole genome duplication (WGD) is just like what it sounds: an event of nondisjunction during meiosis which drives species diversification and evolutionary novelties with additional copies of the entire genome [1–3]. As a common phenomenon in plants, all extant seed plants have experienced at least one ancient WGD, and many flowering plants have undergone multiple rounds of paleopolyploidy [4, 5]. WGD has long been considered as the major force for rapid genome evolution [6–8], which could increase organism complexity, enhance adaptation through dosage effect and induce the speciation and biodiversifcation by immediately producing reproductive isolation with other relatives [9–11]. Moreover, WGD also plays the key role in the domestication of many crops, such as maize, wheat and cotton [12]. For these reasons, there is an increasing interest in detecting the bioinformatic basis of whole genome duplication events.

There are three main methods to search for evidence of WGD [13]. The most straightforward evidence for WGDs is the presence of large syntenic regions within a genome, while these methods need a well-assembled genome, the nearer to chromosome level the more accurate of the results [14, 15]. With a growing number of published draft genomes, two other methods based on phylogenetics [4, 16] and distribution of pairwise paralogs synonymous substitutions per synonymous site (dS) are more suitable [17, 18]. For the former, the WGDs are estimated through the gene count data where the number of gene copies in various gene families across a group of taxa along the phylogeny is counted with the gene birth and death rates in consideration [16]. And the dS based method assumes that each gene family has the constant rates of birth or loss death [19], while WGDs violate this assumption and produce peaks in cumulative distributions of pairwise dS between paralogs within a genome [18]. Recently, the dS based method has become the most common and widely used approach to inferring WGD. Theoretically, peaks in cumulative distributions of pairwise dS between paralogs within the same species should be universal in both genome and transcriptome annotations. Here, we just focused on the dS based method to develop a technical tool for detecting WGDs and trying to break its limitation for utilization of only genome annotations.

Within dS based method, the core and initial step is to identify the pairwise paralogs among the genome, and then, to estimate the distribution of fourfold synonymous third-codon transversion rate (4DTV) or dS between paralogs pairs to determine the WGDs. There are two main approaches to identifying the pairwise paralogs. One is to use the combined gene similarity and gene order information to identify syntenic pairwise paralogs, through many software including ADHoRe [20], DAGchainer [21], ColinearScan [22], MCscan [23], MCScanX [24], SyMAP [25], and so on. The gene order information is unavailable in the poor quality genome assembly or the transcriptomes, which will limit the usage of those software. The other is to use a gene family based approach to identifying pairwise paralogs, which does not need the gene location information and can be suitable for most genomes. However, how to convert the gene family dS to represent the pairwise paralogs dS is complex, since the large gene family need to correct the redundant dS values. Those analyses or approaches are mainly achieved by in-house scripts [18, 26], which are difficult to transfer the same analyses for other species or web servers [27]. Therefore, the repeated attempts are needed, which would cause the wastes of time and resources. To phase those problems, we construct this WGDdetector pipeline for WGDs detecting that integrates all analyses processed with only one command, which includes gene family constructing and dS estimating and phasing, and outputting the dS values of each paralogs pairs.

## Implementation

**Fig. 1:**
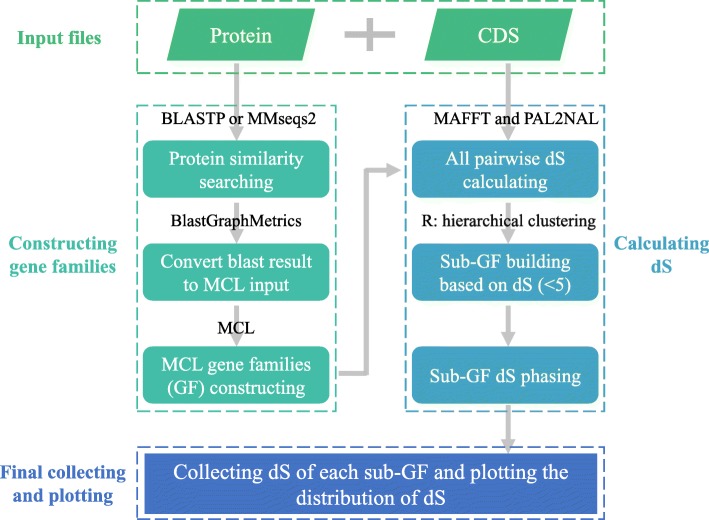
Workflow in WGDdetector. The input files only including the protein and CDS files. The proteins were used in the similarity searching and gene family constructing. The CDSs were used to calculating dS values based on the proteins constructed gene family information. The further sub-gene family building and dS phasing were implemented with the Perl scripts and the R software

WGDdetector is written in Perl. BioPerl must be installed and other seven easily installed software (BLAST [28], MMseqs2 [29], BlastGraphMetrics [30], MCL [31], MAFFT [32], PAL2NAL [33] and R [34]) are also needed. Their function was used in our pipeline and the major steps were exhibited in Fig. 1, and the detailed process was described as follow:

### Gene family constructing

In this step, WGDdetector supplied two methods to detect the gene similarity: BLAST [28] or MMseqs2 [29] with an e-value cut-off of 1e-10. Here, we recommend selecting MMseqs2, as it can run 10,000 times faster than BLAST, and the results were similar. Then the BlastGraphMetrics [30] was used to phase the similarity file, and the followed MCL [31] was selected to construct the gene families based on the Markov Cluster algorithm.

### dS value estimating

WGDdetector automatically aligned the protein and CDS sequence within each gene family using MAFFT [32] and PAL2NAL [33], and assigned the corresponding dS values for each pair paralogs (gap-stripped alignment length > 90 bp) within each gene family via the ‘Bio::Align::DNAStatistics’ Perl module based on the Nei-Gojobori algorithm.

### dS correction for redundant

As the above estimates, a gene family of *n* members originated from *n-1* retained single gene duplications and generated the number of possible pairwise comparisons is *n(n-1)/2*. To correct the redundancy of dS values, we used a slightly modified strategy as described in *Arabidopsis* [18] and Norway spruce analysis [35]. We used the dS as a distance measure, and constructed a tentative phylogenetic tree with an average linkage clustering algorithm using the ‘hclust’ R module. A series of clusters (from 1 to *n*, *n* is the gene numbers within one family) were generated by the ‘cutree’ function for each gene family. Subsequently, they were divided into the subfamilies with the dS values less than 5 and each subfamily contained as many genes as possible. Then, a tentative phylogenetic tree was constructed again for each subfamily, and ‘cutree’ was used to intercept only two child clades. We summed the dS values for all combinations between the two child clades, and weighed the number of combinations to represent this subfamily, which corresponded to a duplication event. Finally, we collected all the dS values of each subfamily and supply the R script to plot the distribution.

## Results

Four organisms’ genome or/and transcriptome datasets were selected to evaluate the performance of WGDdetector, including three plants (*Arabidopsis thaliana*, *Juglans regia* and *Populus trichocarpa*) and one frog (*Xenopus laevis*) (Table 1 and Additional file 1: Table S1). For the genome datasets, a total of 27,301, 32,436, 39,410 and 41,073 genes satisfied our criteria in *A. thaliana*, *J. regia*, *P. trichocarpa* and *X. laevis*, respectively: retaining the longest coding sequence (CDS) for each gene, removing CDS with premature stop codons and those protein sequences < 50 amino acids (AA). For the transcriptome datasets, the raw reads were download from NCBI SRA and assembled by Trinity v2.5.1 [36] with the default parameters except “--trimmomatic” and “--normalize_reads”. The constructed transcripts were filtered by the SeqClean [37] to remove contamination, and then the TransDecoder v5.3.0 [38] was used to identify candidate coding regions. The candidate alternative splicing were filtered by CD-HIT-EST with the parameter ‘-c 0.9′ [39], and the proteins with length less than 50 AA were further removed. A total of 23,495 and 20,354 transcripts were obtained for the following analysis in *A. thaliana* and *P. trichocarpa*, respectively.

**Table 1:** Statistics of the WGDdetector performance on the test data

Software	Date type	Clean genes	Number of threads^a^	Elapsed time (hour)^b^	Max memory used (Gb)	Number ofsub-gene families^c^
ADHoRe	*A. thaliana* (genome)	27,301	15; 1	1.79 (1.05 + 0.74)	0.94	6649
	*J. regia* (genome)	32,436	15; 1	2.09 (1.23 + 0.86)	2.11	6127
	*P. trichocarpa* (genome)	39,410	15; 1	4.18 (1.51 + 2.67)	2.52	22,676
	*X. laevis* (genome)	41,073	15; 1	4.82 (2.24 + 2.58)	4.08	14,055
MCScanX	*A. thaliana* (genome)	27,301	15; 1	1.62 (1.05 + 0.57)	0.73	14,363
	*J. regia* (genome)	32,436	15; 1	2.64 (1.23 + 1.41)	1.44	4333
	*P. trichocarpa* (genome)	39,410	15; 1	2.21 (1.51 + 0.70)	1.56	21,643
	*X. laevis* (genome)	41,073	15; 1	6.27 (2.24 + 4.03)	1.65	23,721
WGDdetector	*A. thaliana* (genome)	27,301	15	5.57	9.16	6729
	*A. thaliana* (RNA-seq)	23,495	15	6.32	6.32	6072
	*J. regia* (genome)	32,436	15	8.88	15.56	8504
	*P. trichocarpa* (genome)	39,410	15	11.65	11.65	9818
	*P. trichocarpa* (RNA-seq)	20,354	15	5.18	11.84	5191
	*X. laevis* (genome)	41,073	15	19.23	35.46	9391

^a^The format of “15; 1” representing the number of threads when protein clustering and the dS calculating

^b^The format “S (X + Y)” represent the total elapsed time (S), the protein clustering elapsed time (X) and the dS calculating elapsed time (Y)

^c^In the software ADHoRe and MCScanX, all the homologous genes were recorded. In the WGDdetector pipeline, only the sub-gene families were recoded

**Fig. 2:**
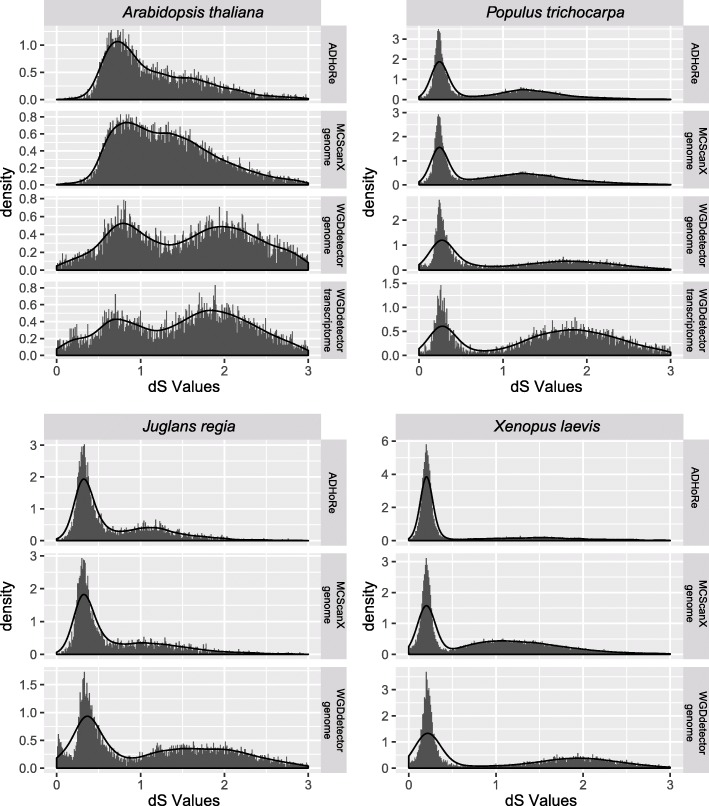
Comparison of the dS distributions within *A. thaliana* and *P. trichocarpa*. The y axis is the density values and the x axis represent the dS values. The four species were marked at the top of each sub picture. The software and corresponding dataset were listed at the right of each sub picture

All datasets with a gene number ranging from 20,354 to 41,073, showed a memory usage approximately 6~35G and the elapsed time around 5-19 h (Table 1). As our pipeline could use multiple CPUs, this elapsed time would be shorter if more CPUs used. To evaluate the performance of WGDdetector, ADHoRe and MCScanX were selected for comparisons. The general similar trajectories of the density or histogram were observed in all the datasets implemented by WGDdetector, ADHoRe and MCScanX, and different software have different superiority at the recent or ancient WGD events (Fig. 2). All the first peaks were coincidence by different approaches within each species, which indicated high sensitivity and accuracy in the detection of recent WGD event using WGDdetector, based on both genome and transcriptome datasets. For *A. thaliana*, a major peak (the second) with a long range (0.7~2) was detected using both ADHoRe and MCScanX, which was hard to discern the ancient WGD event. While, the result of WGDdetector showed two peaks (~ 0.6 and ~ 1.9), representing the 1R and 2R WGD events within *A. thaliana* and coincident with the previous studies [18, 40]. In the other three species, WGDdetector also showed a high sensitive on the detection of ancient WGD event, as a more obvious second peak detected than the other two software. But we also found slightly larger dS values in the second peak in WGDdetector than the other software, as detected in *P. trichocarpa* (ADHoRe: ~ 1.3, MCScanX: ~ 1.4, WGDdetector: ~ 1.7), *J. regia* (ADHoRe: ~ 1.3, MCScanX: ~ 1.2, WGDdetector: ~ 1.5) and *X. laevis* (ADHoRe: ~ 1.5, MCScanX: ~ 1.1, WGDdetector: ~ 1.8).

## Discussion

As the methodological distinctness at the dS distribution obtaining, WGDdetector elapsed more time and memory than ADHoRe and MCScanX (Table 1). This was mainly caused by the most time consuming step that WGDdetector calculated the dS values between all the possible homologous gene pairs within each gene family. While ADHoRe and MCScanX needed the gene order information to identify the synteny gene pairs and thereby a small number of dS values were calculated [24]. In the results of accuracy evaluation, WGDdetector showed a high accuracy and more sensitive of detecting the recent WGD events. In the genome data of *J. regia* or the transcriptome data of *A. thaliana* and *P. trichocarpa*, WGDdetector was also detected noise signal peaks (near the origin), which might reflect the unmerged allelic haplotypes in the genome data [41] or the alternative splice transcripts within the transcriptome data that was still retained. Our results of the genome data of *A. thaliana* also proved the distinct first and second peaks, rather than a long range peak detected in MCScanX and ADHoRe, which reflecting the high performance of detecting the ancient WGD events in WGDdetector. The second peaks in each dataset showed a little difference in different software. We speculated that this difference might be caused by dS saturation when the dS value > 1 [42], and the higher sensitive performance in the detecting ancient WGD in WGDdetector than that in ADHoRe and MCScanX.

## Conclusions

The WGDdetector was designed as a user-friendly pipeline with a very simple command which only needed the CDS and protein files. This pipeline integrated the gene family constructing, dS estimating and hierarchical clustering, dS correcting and distributing plotting. This methodology eliminates the limitation of gene order information and is more suitable for the well/poor quality genomes and transcriptomes. In our practice based on the genome and transcriptome datasets, WGDdetector showed a high performance in the detection of recent and ancient WGD events and matched well with the previous studies and/or the software of ADHoRe and MCScanX. With the development and rapidly declining cost of next-generation sequencing (NGS) technologies and third-generation long-range DNA sequencing, more and more species would be resolved by sequencing their genomes and/or transcriptomes [43, 44]. Totally, WGDdetector gives a reliable and acceptable way to infer WGD event using either genome or transcriptome data by the dS-based method, and will help to accelerate our understanding of the evolutionary history of WGDs within all organisms.

### Availability and requirements

Project name: WGDdetector.

Project home page: https://github.com/yongzhiyang2012/wgddetector

Operating system(s): Windows and Linux.

Programming language: Perl, R.

Other requirements: Python 2.7, parallel, MMseqs2, BLAST, BlastGraphMetrics, mcl, MAFFT and PAL2NAL.

License: GNU GPL v3.

Any restrictions to use by non-academics: none.

## Additional file

Additional file 1: **Table S1.** Data used during the WGD analysis. (DOCX 14 kb)

## References

[1] del Pozo JC, Ramirez-Parra E (2015). Whole genome duplications in plants: an overview from Arabidopsis. J Exp Bot.

[2] Van de Peer Y, Mizrachi E, Marchal K (2017). The evolutionary significance of polyploidy. Nat Rev Genet.

[3] Wendel JF (2015). The wondrous cycles of polyploidy in plants. Am J Bot.

[4] Jiao Y, Wickett NJ, Ayyampalayam S, Chanderbali AS, Landherr L, Ralph PE, Tomsho LP, Hu Y, Liang H, Soltis PS (2011). Ancestral polyploidy in seed plants and angiosperms. Nature.

[5] Li Z, Baniaga AE, Sessa EB, Scascitelli M, Graham SW, Rieseberg LH, Barker MS (2015). Early genome duplications in conifers and other seed plants. Sci Adv.

[6] Jackson S, Chen ZJ (2010). Genomic and expression plasticity of polyploidy. Curr Opin Plant Biol.

[7] Ramsey J, Schemske DW (2002). Neopolyploidy in flowering plants. Annu Rev Ecol Syst.

[8] Wendel JF (2000). Genome evolution in polyploids. Plant Mol Biol.

[9] Arrigo N, Barker MS (2012). Rarely successful polyploids and their legacy in plant genomes. Curr Opin Plant Biol.

[10] Gout JF, Lynch M (2015). Maintenance and loss of duplicated genes by dosage subfunctionalization. Mol Biol Evol.

[11] Soltis DE, Visger CJ, Soltis PS (2014). The polyploidy revolution then… and now: Stebbins revisited. Am J Bot.

[12] Dubcovsky J, Dvorak J (2007). Genome plasticity a key factor in the success of polyploid wheat under domestication. Science.

[13] Tiley GP, Ane C, Burleigh JG (2016). Evaluating and characterizing ancient whole-genome duplications in plants with gene count data. Genome Biol Evol.

[14] Conant GC (2014). Comparative genomics as a time machine: how relative gene dosage and metabolic requirements shaped the time-dependent resolution of yeast polyploidy. Mol Biol Evol.

[15] Jaillon O, Aury JM, Brunet F, Petit JL, Stange-Thomann N, Mauceli E, Bouneau L, Fischer C, Ozouf-Costaz C, Bernot A (2004). Genome duplication in the teleost fish Tetraodon nigroviridis reveals the early vertebrate proto-karyotype. Nature.

[16] Rabier CE, Ta T, Ane C (2014). Detecting and locating whole genome duplications on a phylogeny: a probabilistic approach. Mol Biol Evol.

[17] Barker MS, Kane NC, Matvienko M, Kozik A, Michelmore RW, Knapp SJ, Rieseberg LH (2008). Multiple paleopolyploidizations during the evolution of the Compositae reveal parallel patterns of duplicate gene retention after millions of years. Mol Biol Evol.

[18] Maere S, De Bodt S, Raes J, Casneuf T, Van Montagu M, Kuiper M, Van de Peer Y (2005). Modeling gene and genome duplications in eukaryotes. Proc Natl Acad Sci U S A.

[19] Raes J, Vandepoele K, Simillion C, Saeys Y, Van de Peer Y (2003). Investigating ancient duplication events in the Arabidopsis genome. J Struct Funct Genom.

[20] Proost S, Fostier J, De Witte D, Dhoedt B, Demeester P, Van de Peer Y, Vandepoele K (2012). I-ADHoRe 3.0--fast and sensitive detection of genomic homology in extremely large data sets. Nucleic Acids Res.

[21] Haas BJ, Delcher AL, Wortman JR, Salzberg SL (2004). DAGchainer: a tool for mining segmental genome duplications and synteny. Bioinformatics.

[22] Wang X, Shi X, Li Z, Zhu Q, Kong L, Tang W, Ge S, Luo J (2006). Statistical inference of chromosomal homology based on gene colinearity and applications to Arabidopsis and rice. BMC Bioinf.

[23] Tang H, Wang X, Bowers JE, Ming R, Alam M, Paterson AH. Unraveling ancient hexaploidy through multiply-aligned angiosperm gene maps. Genome Res. 2008;18(12):1944–54. 10.1101/gr.080978.108 PMC2593578 18832442

[24] Wang Y, Tang H, Debarry JD, Tan X, Li J, Wang X, Lee TH, Jin H, Marler B, Guo H (2012). MCScanX: a toolkit for detection and evolutionary analysis of gene synteny and collinearity. Nucleic Acids Res.

[25] Soderlund C, Nelson W, Shoemaker A, Paterson A (2006). SyMAP: a system for discovering and viewing syntenic regions of FPC maps. Genome Res.

[26] Vanneste K, Baele G, Maere S, Van de Peer Y (2014). Analysis of 41 plant genomes supports a wave of successful genome duplications in association with the cretaceous-Paleogene boundary. Genome Res.

[27] Barker MS, Dlugosch KM, Dinh L, Challa RS, Kane NC, King MG, Rieseberg LH (2010). EvoPipes.net: Bioinformatic tools for ecological and evolutionary genomics. Evol Bioinformatics Online.

[28] Camacho C, Coulouris G, Avagyan V, Ma N, Papadopoulos J, Bealer K, Madden TL (2009). BLAST+: architecture and applications. BMC Bioinf.

[29] Steinegger M, Soding J (2017). MMseqs2 enables sensitive protein sequence searching for the analysis of massive data sets. Nat Biotechnol.

[30] Gibbons TR, Mount SM, Cooper ED, Delwiche CF (2015). Evaluation of BLAST-based edge-weighting metrics used for homology inference with the Markov clustering algorithm. BMC Bioinf.

[31] Van Dongen SM. Graph clustering by flow. SIMULATION. 2000.

[32] Katoh K, Standley DM (2013). MAFFT multiple sequence alignment software version 7: improvements in performance and usability. Mol Biol Evol.

[33] Suyama M, Torrents D, Bork P (2006). PAL2NAL: robust conversion of protein sequence alignments into the corresponding codon alignments. Nucleic Acids Res.

[34] Team RC. R: a language and environment for statistical. Computing. 2013.

[35] Nystedt B, Street NR, Wetterbom A, Zuccolo A, Lin YC, Scofield DG, Vezzi F, Delhomme N, Giacomello S, Alexeyenko A (2013). The Norway spruce genome sequence and conifer genome evolution. Nature.

[36] Grabherr MG, Haas BJ, Yassour M, Levin JZ, Thompson DA, Amit I, Adiconis X, Fan L, Raychowdhury R, Zeng Q (2011). Full-length transcriptome assembly from RNA-Seq data without a reference genome. Nat Biotechnol.

[37] SeqClean https://sourceforge.net/projects/seqclean/.

[38] Haas B, Papanicolaou A: Transdecoder (Find Coding Regions within Transcripts).; 2012 http://transdecoder.sourceforge.net.

[39] Li W, Godzik A (2006). Cd-hit: a fast program for clustering and comparing large sets of protein or nucleotide sequences. Bioinformatics.

[40] Blanc G, Hokamp K, Wolfe KH (2003). A recent polyploidy superimposed on older large-scale duplications in the Arabidopsis genome. Genome Res.

[41] Martinez-Garcia PJ, Crepeau MW, Puiu D, Gonzalez-Ibeas D, Whalen J, Stevens KA, Paul R, Butterfield TS, Britton MT, Reagan RL (2016). The walnut (Juglans regia) genome sequence reveals diversity in genes coding for the biosynthesis of non-structural polyphenols. Plant J.

[42] Vanneste K, Van de Peer Y, Maere S (2013). Inference of genome duplications from age distributions revisited. Mol Biol Evol.

[43] Kulski JK. Next-generation sequencing-an overview of the history, tools, and “Omic” applications. In: Next generation sequencing-advances, applications and challenges. IntechOpen. 2016. 10.5772/61964. Available from: https://www.intechopen.com/books/next-generation-sequencing-advances-applications-and-challenges/next-generation-sequencing-an-overview-of-the-history-tools-and-omic-applications.

[44] Lee H, Gurtowski J, Yoo S, Nattestad M, Marcus S, Goodwin S, McCombie WR, Schatz M. Third-generation sequencing and the future of genomics. BioRxiv. 2016;048603.

