# Supplementary material for: WGDdetector: a pipeline for detecting whole genome duplication events using the genome or transcriptome annotations
Source: BMC Bioinformatics. 2019 Feb 13;20:75. doi: 10.1186/s12859-019-2670-3 (PMC6375192; doi:10.1186/s12859-019-2670-3)
Supplement: Supplementary file 1 — Table S1. Data used during the WGD analysis. (DOCX 14 kb) [file 12859_2019_2670_MOESM1_ESM.docx]

**Table S1. Data used during the WGD analysis**

| **Species** | **Data type** | **Accesion number** | **Database** | **Original genes^1^** | **Clean genes^2^** | **N50 length of clean CDS^3^** |
| --- | --- | --- | --- | --- | --- | --- |
| *Arabidopsis thaliana* | genome | TAIR10 | EnsemblPlants 40 | 27,655 | 27,301 | 1,533 |
|  | transcriptome | SRR7594348 | NCBI SRA | 64,556 | 23,495 | 1,407 |
| *Populus trichocarpa* | genome | Pop_tri_v3 | EnsemblPlants 40 | 41,335 | 39,410 | 1,545 |
|  | transcriptome | SRR3019937 | NCBI SRA | 58,685 | 20,354 | 1,536 |
| *Juglans regia* | genome | PRJNA291087 | NCBI | 32,496 | 32,436 | 1,554 |
| *Xenopus laevis* | genome | X. laevis v9.2 | http://www.xenbase.org | 41,374 | 41,073 | 2,022 |

^1^Original genes represent the raw genes number of the genome annotation or the raw transcripts number of Trinity assembly results.

^2^Clean genes represent the final cleaned gene used to detector the WGDs. Genome data filtering criteria: retain the longest transcripts (larger than 50 AA) for each gene and removed genes with ambiguous relationship between the CDS and proteins. Transcriptome data filtering criteria: retain the transcripts filtered by SeqClean and CD-HIT-EST, and removed the proteins with a length less than 50 AA.

^3^N50 refers to the size above which 50% of the total length of CDS can be found.
